# Effectiveness of gilt acclimatization – improvement procedures in a farm with recurrent outbreaks of porcine epidemic diarrhea

**DOI:** 10.14202/vetworld.2023.1695-1701

**Published:** 2023-08-24

**Authors:** Pimpakarn Suwan, Alongkot Boonsoongnern, Sahathat Phuttapatimok, Manakorn Sukmak, Pichai Jirawattanapong, Wilairat Chumsing, Orawan Boodde, Krithiran Woramahatthanon, Yonlayong Woonwong

**Affiliations:** 1 Graduate Program in Veterinary Clinical Studies, Faculty of Veterinary Medicine, Kasetsart University, Kamphaeng Saen, Nakhon Pathom, 73140, Thailand; 2 Department of Farm Resources and Production Medicine, Faculty of Veterinary Medicine, Kasetsart University, Kamphaeng Saen, Nakhon Pathom, 73140, Thailand; 3 Kamphaengsaen Veterinary Diagnostic Center, Faculty of Veterinary Medicine, Kasetsart University, Kamphaeng Saen, Nakhon Pathom, 73140, Thailand

**Keywords:** acclimatization, gilt, immune response, phylogenetic tree, porcine epidemic diarrhea

## Abstract

**Background and Aim:**

Porcine epidemic diarrhea (PED) is a severe infectious disease that causes very high mortality in newborn piglets up to 2–3 weeks age. The main cause of repeated outbreaks of PED in infected farms is the continuing circulation of the PED virus (PEDV). Improper gilt management, including inappropriate gut feedback, commingling, and inadequate immunization, causes a prolonged virus circulation in breeding herds. Moreover, insufficient transfer of passive immunity through the colostrum to newborn piglets can also increase infection risk. Therefore, a gilt management program that controls infection should focus on infection monitoring and acclimatization. We investigated the source of recurrent PEDV outbreaks and examined how the effect of immunization methods, specifically using gut feedback mechanism and vaccination, can reduce PEDV circulation and improve immune responses in replacement gilts.

**Materials and Methods:**

The study site was a segregated commercial production farm with endemic PEDV. The acclimatization methods included gut feedback and vaccination. This longitudinal study evaluated two strategies of gilt acclimatization against PEDV: Program 1 (routine farm management) and Program 2 (early feedback program and all-in-all-out system). Levels of PED RNA in fecal samples were measured using quantitative reverse transcription-polymerase chain reaction, and the PEDV S gene was sequenced. Porcine epidemic diarrhea-specific immune responses were assessed using enzyme-linked immunosorbent assay and the serum neutralization test.

**Results:**

Porcine epidemic diarrhea outbreaks occurred in the farrowing, nursery, and finishing units and farrowed litters 5–10 days old were symptomatic of PED. Phylogenetic analyses of the S gene showed PEDV sequence divergence between PEDV field strains and vaccine strain, which may contribute to periodic outbreaks and continued persistence of PEDV in the farm. After gut feedback and acclimatization, replacement gilts from Program 1 continued to shed PEDV before being introduced to sow herds, while those from Program 2 did not shed PEDV before being introduced to sow herds. However, the components of the immune response against PEDV in serum samples, including specific immunoglobulin (Ig)G, specific IgA, and neutralizing antibodies were lower in gilts of Program 2 than those in Program 1.

**Conclusion:**

We speculate that implementing the appropriate gilt acclimatization program can control PEDV circulation in farm. However, the acclimatization methods in Program 2 did not induce a strong and adequate immune response in replacement gilts. Therefore, maternal immunity levels and the degree of protection against PEDV require further study.

## Introduction

In the past decade, porcine epidemic diarrhea (PED) has become one of the most severe infectious diseases that cause large-scale economic losses in pig production. Infections caused by the PED virus (PEDV) cause severe diarrhea, vomiting, and dehydration, leading to a very high mortality rate in suckling pigs. However, older pigs may develop only mild diarrhea and are often virus carriers, resulting in endemicity [1]. Before 2007, most of the PEDV strains identified in Thailand were classical strains, and their outbreaks occurred only in the southern region of Thailand [2]. In late 2007, a new PEDV strain variant was reported in Thailand [3] and this variant has continued to spread across the country. It is now considered an endemic pathogen that causes “reoutbreak” of PEDV in infected farms [4].

The recurrent outbreaks of PED in infected herds are due to the continuous circulation of PEDV in the environment, which constitutes the main risk factor for this disease [5, 6]. Therefore, infection control requires an appropriate program focusing on infection monitoring, biosecurity management, and acclimatization [7]. The gut mucosal immune system plays a role in protective immunity against PEDV infection [8] and the main cause of PED outbreaks among newborn piglets is the inadequate transfer of passive immunity from inappropriately immunized sows with PEDV vaccine and/or through the gut feedback mechanism to the newborn piglets [9]. Therefore, monitoring and managing sow health status and protective immunity against PEDV infection is crucial to ensure adequate transfer of high immunity levels through the colostrum to newborn piglets. An effective gilt acclimatization program that improves immunity in newborn piglets through the colostrum is one of the major approaches that can reduce the risk of PED outbreaks in PED-endemic herds [10]. However, improper gilt management may lead to PEDV persistence in the sow herd. Soon after the gut feedback and commingling procedures are implemented during acclimatization program, the replacement gilts become infected and may persistently shed the virus. This causes prolonged circulation (up to 69 days after PEDV exposure) of PEDV within the breeding herd at the pen level [11], thus resulting in “reoutbreak” in the farrowing unit [12].

Therefore, to control PEDV circulation in the endemic herd, an appropriate gilt management program that focuses on acclimatization and immunity improvement is urgently needed.

This study aimed to investigate how gut feedback mechanism and PEDV vaccination in gilt development unit can affect PEDV circulation and improve the immune response of replacement gilts.

## Materials and Methods

### Ethical approval

The study protocol was approved by the Animal Care and Use Committee, Kasetsart University (IACUC #ACKU65-VET-025), and the owner’s approval and agreement to participate in this study were obtained.

### Study period and location

The study was conducted from June 2020 to December 2021 in a commercial swine herd in Thailand that experienced recurrent outbreaks of PED. The samples were processed at the Kamphaeng Saen Veterinary Diagnostic Center, Faculty of Veterinary Medicine, Kasetsart University.

### Farm characteristics

The study site was a P0-P1 segregated commercial production farm with endemic PEDV. The farm had approximately 5000 sows in four units (700–1200 sows in each unit). All replacement gilts (Large White × Landrace) were internally replaced at 12 weeks of age (20 pigs per pen). The replacement gilts were divided into three groups according to the phase of the gilt management program and were housed in three barns: The nursery to grower barn (G1), finisher barn (G2), and the replacement gilt barn (G3). All gilt management barns had an evaporative cooling system in the semi-isolated area. The G1 and G2 barns had 20 pens on both sides of the walkway, whereas the G3 barn had 100 individual stalls at the back of the barn. Incoming gilts were immunized against PED by two methods: (i) PEDV inactivated vaccine (SuiShot^®^ PT-100, ChoongAng Vaccine Laboratories Co., Ltd. (CAVAC), Daejeon, South Korea) and (ii) acclimatization using the intestinal contents from piglets with PED (gut feedback). The gilts were also vaccinated against porcine reproductive and respiratory syndrome virus, porcine circovirus type 2 viruses, foot-and-mouth disease virus, and classical swine fever virus, Aujeszky’s disease virus, porcine parvovirus, *Erysipelothrix rhusiopathiae*, leptospirosis, and *Actinobacillus pleuropneumoniae*.

### Experimental design

**Figure-1:**
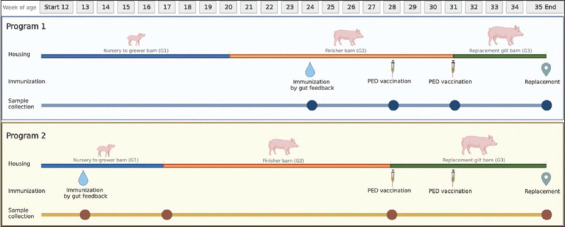
Experimental design in Program 1 and Program 2; including pigflow management, immunization program and sample collection.

To study the acclimatization process for PEDV infection status, we conducted a longitudinal study to evaluate two acclimatization methods (Programs 1 and 2). In both methods, gilts were immunized against PEDV through gut feedback and vaccination. The experimental design of each program is illustrated in Figure-1. Before initiating the experiment, the gilts were immunized through “Program 1,” which involved PEDV feedback at 24-week-old in a G2 barn through minced intestinal tissues of PEDV-infected piglets. In addition, the gilts received intramuscular vaccinations of PEDV vaccine when they were 28 and 31 weeks old. After the 31-week-old vaccination, the gilts were moved to individual stalls in the G3 barn for the cool-down period.

In “Program 2,” the acclimatization method involved implementation of PEDV gut feedback in the G1 barn when the gilts were 13 weeks old. These gilts then received intramuscular vaccinations against PEDV when they were 28 and 31 weeks old (both in the G2 barn). The 28-week-old vaccinated gilts were moved to individual stalls in the G3 barn for the cool-down period. In both programs, samples were collected using similar procedures, with minor differences. The statuses of their viral kinetics, and immune responses, were compared until gilt replacement. Moreover, porcine epidemic diarrhea virus infections among pigs were recorded, especially when animals exhibited clinical signs of PED. Porcine epidemic diarrhea virus S gene sequences were confirmed through quantitative reverse transcription-polymerase chain reaction (RT-qPCR).

### Sample collection

Five pigs from four pens (containing 20 immunized gilts) from each program were selected for serial sample collection. Sample collection started on the day of acclimatization and ended at replacement. Blood and fecal samples were collected at each acclimatization period: (i) Before immunization by gut feedback, (ii) 4 weeks after immunization, (iii) during the cool-down period, and (iv) during replacement. Samples were collected from Program 1 gilts at 24, 28, 31, and 35 weeks of age and from Program 2 gilts at 13, 17, 28, and 35 weeks of age. Blood samples (5 ml) were centrifuged at 8000x g for 5 min and the resulting sera were aliquoted into microcentrifuge tubes. Fecal samples were collected using cotton swabs, which were then transferred to 500 mL of D-solution (Himedia, India). The solution and samples were mixed thoroughly by vortex mixing and stored at −80°C until testing.

### Virus detection, quantification, and sequencing of PEDV

Porcine epidemic diarrhea virus was detected by RT-qPCR using primers that targeted the PEDV M gene [13]. Total RNA was extracted from all fecal samples according to the modified phenol-chloroform method [14]. Complementary DNA (cDNA) was generated from RNA template using the RevertAid™ First Strand cDNA Synthesis Kit (Thermo Fisher Scientific Baltics UAB, Vilnius, Lithuania), according to the manufacturer’s instructions. The cDNA was tested for the presence of PEDV nucleic acids using a TaqMan^®^ probe (Biolab Co., Ltd., Samutrprakarn, Thailand) [13]. The RT-qPCR assay was performed using a Bio-Rad CFX96 RT-PCR Detection System. The limit of detection of the assay was set at a Ct value of ≥36 to assign a negative result for the PEDV RT-qPCR assay.

Nucleotide sequences of the partial spike gene from PEDV-positive samples were evaluated by first aligning them using BioEdit version 7.2.6.1 [15] and MEGA11 [16] and then assessing the phylogenetic relationships between PEDV strains from this study and those that had been previously identified by Tuanthap *et al*. [6].

### Serological examinations

All serum samples were submitted to the Kamphaeng Saen Veterinary Diagnostic Center, Faculty of Veterinary Medicine, Kasetsart University. The serum samples were analyzed using a whole-virus enzyme-linked immunosorbent assay designed to detect the levels of specific immunoglobulin (Ig)G and specific IgA of PEDV [17]. An S/P ratio of >0.4 was considered a PEDV Ab-positive sample. The serum neutralization (SN) test was performed to detect neutralizing antibodies against PEDV, as previously described by Baek *et al*. [9], with minor modifications. The serum samples were prepared by heat inactivation at 56°C for 30 min, followed by two-fold serial dilution of the serum sample in a 96-well plate (50 ml/well) and incubated with 50 ml of the virus solution (100 TCID_50_/well). The plates were incubated at 37°C for 1 h. The serum-virus mixtures were transferred into Vero cells and incubation at 37°C for 48 h. The SN titer was defined as the highest dilution of the serum that inhibited the cytopathic effects (CPE).

### Statistical analysis

Statistical analyses were performed using R Studio version 4.0.1 (https://posit.co/download/rstudio-desktop/). The viral titers, S/P ratios, and neutralizing antibodies associated with Program 1 and Program 2 were compared at each time point using independent t-tests. The frequencies of virus-positive animals were compared among the groups using Fisher’s exact test. p < 0.05 was considered statistically significant.

## Results

**Figure-2:**
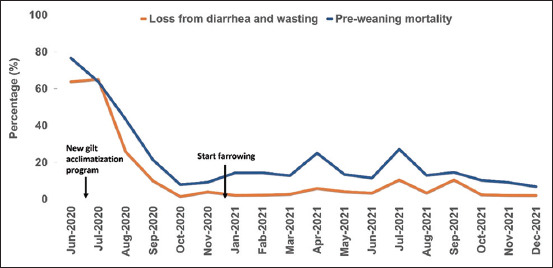
Mortality rate in piglets following the porcine epidemic diarrhea virus outbreak in farrowing unit during June 2020–December 2021.

The numbers of suckling piglets lost due to diarrhea and wasting from June 2020 to December 2021 are shown in Figure-2. Endemic outbreaks of PED occurred in the farrowing, nursery, and finishing units from June 2020 to September 2020. Farrowing unit litters usually showed clinical signs of PED during 5–10 days of age. The mortality rate of newborn piglets in the farrowing unit dropped significantly in August 2020, and pig production gradually returned to a stable rate. In the nursery unit, some pigs also had diarrhea and wasting (5%–10%), while 5% of finishing pigs had mushy diarrhea and recovered in a few days. Moreover, the source of the replacement gilts was PED-positive based on fecal analysis.

**Figure-3:**
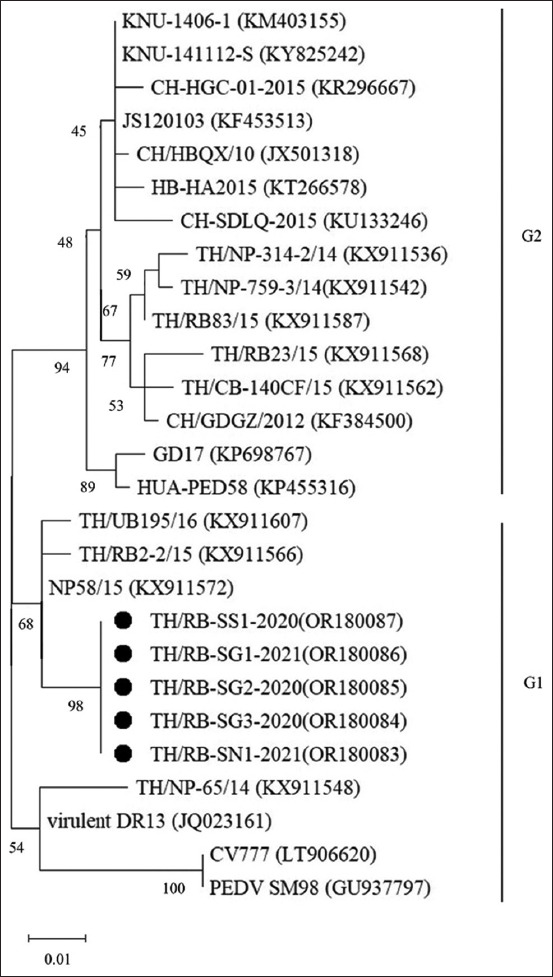
Phylogenetic analysis of porcine epidemic diarrhea virus based on the nucleotide sequences of the partial S glycoprotein genes. The tree was constructed using the maximum likelihood method with the Hasegawa-Kishino-Yano model and bootstrap re-sampling (5000 replications). The black circle represents the intra-farm culture sample.

The sequences of the partial S gene in five PEDV-positive samples collected from affected pigs were analyzed. Phylogenetic analysis showed the S gene sequences were clustered into two major groups: G1 and G2 (Figure-3). All PEDV strains from this study were 100% identical and clustered in the G1 group. These PEDV strains are closely related to some previously identified Thai strains’ accession number KX911572, KX911566, and KX911607, and they share 94% identity to the prototypic strain CV777 and vaccine strains (GU937797).

The dynamics of PEDV shedding in the replacement gilts of Programs 1 and 2 are shown in Table-1. Quantitative reverse transcription-polymerase chain reaction results indicate fecal PEDV shedding in both groups at the start of the programs. Porcine epidemic diarrhea virus shedding in the Program 1 group occurred throughout the acclimatization period; four replacement gilts were positive for PEDV at 35 weeks of age. In contrast, all Program 2 gilts did not shed PEDV in their feces at either 28 or 35 weeks of age.

**Table-1:** The results of individual fecal samples testing positive for PEDV by RT-qPCR in Program 1 and Program 2 by age of pigs (weeks)*.

Program	Pig no.	Detection of PEDV using RT-qPCR by age of pigs (weeks)^†^					

		13	17	24	28	31	35
1	1	NA	NA	-	-	+	-
	2	NA	NA	-	+	+	+
	3	NA	NA	-	-	-	-
	4	NA	NA	-	-	-	-
	5	NA	NA	-	+	+	-
	6	NA	NA	+	+	-	-
	7	NA	NA	-	+	-	+
	8	NA	NA	-	+	+	+
	9	NA	NA	-	-	-	-
	10	NA	NA	-	+	+	-
	11	NA	NA	-	-	-	-
	12	NA	NA	-	-	-	-
	13	NA	NA	-	+	-	-
	14	NA	NA	-	+	+	-
	15	NA	NA	-	+	-	-
	16	NA	NA	-	-	-	-
	17	NA	NA	-	-	-	-
	18	NA	NA	+	-	+	-
	19	NA	NA	+	-	-	-
	20	NA	NA	-	+	-	+
No. of positive pigs		NA	NA	3/20	10/20	7/20	4/20
2	1	-	-	NA	-	NA	-
	2	+	+	NA	-	NA	-
	3	-	-	NA	-	NA	-
	4	-	-	NA	-	NA	-
	5	-	-	NA	-	NA	-
	6	-	-	NA	-	NA	-
	7	+	-	NA	-	NA	-
	8	-	-	NA	-	NA	-
	9	-	-	NA	-	NA	-
	10	+	-	NA	-	NA	-
	11	-	-	NA	-	NA	-
	12	-	-	NA	-	NA	-
	13	+	-	NA	-	NA	-
	14	-	-	NA	-	NA	-
	15	-	-	NA	-	NA	-
	16	-	-	NA	-	NA	-
	17	-	+	NA	-	NA	-
	18	+	-	NA	-	NA	-
	19	-	+	NA	-	NA	-
	20	-	-	NA	-	NA	-
No. of positive pigs		5/20	3/20	NA	0/20	NA	0/20

† The Ct value of PEDV RT-qPCR ≥ 36 was considered as negative results.

* NA=Not available, RT-qPCR=Reverse transcription-quantitative polymerase chain reaction, PEDV=Porcine epidemic diarrhea virus

**Figure-4:**
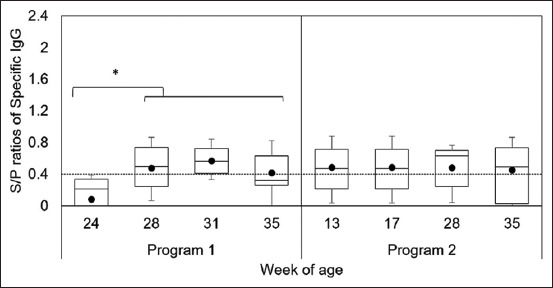
The results of specific immunoglobulin G antibodies against porcine epidemic diarrhea virus in Program 1 and Program 2; *indicates difference (p < 0.05) between the before and after immunization.

The results of assays detecting specific IgG against PEDV are shown in Figure-4. The S/P ratio of gilts in Program 1 was significantly higher after immunization than before immunization (p = 0.00078). Anti-PEDV antibodies were detected in 5%, 55%, 80%, and 45% of Program 1 gilts at 24, 28, 31, and 35 weeks of age, respectively. In contrast, anti-PEDV IgG antibodies were detected in 55%, 60%, 55%, and 55% of Program 2 gilts at 13, 17, 28, and 35 weeks of age, respectively. However, we observed no differences in the S/P ratios of specific IgG antibodies in Program 2 gilts during the monitoring period.

**Figure-5:**
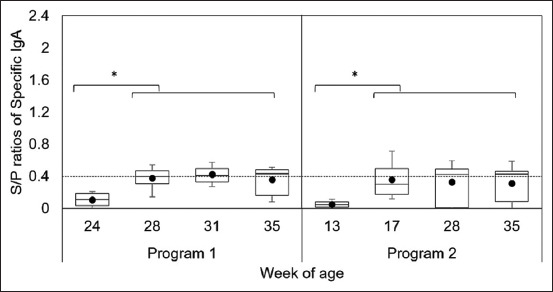
The results of specific immunoglobulin A antibodies against porcine epidemic diarrhea virus in Program 1 and Program 2; *indicates difference (p < 0.05) between the before and after immunization.

The levels of PEDV IgA antibodies are shown in Figure-5. Immunized gilts in Program 1 had a significantly higher S/P ratio than before immunization (p = 0.000028). Porcine epidemic diarrhea virus IgA antibodies were detected in 0%, 50%, 55%, and 60% of immunized gilts at 24, 28, 31, and 35 weeks of age, respectively, while in Program 2 gilts, specific IgA levels were significantly higher after stimulation (p = 0.0045). Porcine epidemic diarrhea virus IgA antibody levels were detected in 0%, 30%, 50%, and 50% of immunized gilts at 13, 17, 28, and 35 weeks of age, respectively.

**Figure-6:**
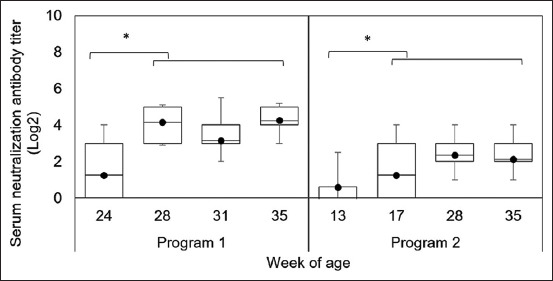
The results of neutralizing antibody titers in Program 1 and Program 2; *indicates difference (p < 0.05) between the before and after immunization.

The levels of serum neutralization (SN) antibodies (Figure-6) show that the levels of SN antibody titers in Program 1 gilts were significantly higher than those before immunization (p = 0.0001). The percentages of Program 1 gilts positive for SN antibodies were 35%, 100%, 80%, and 100% at 24, 28, 31, and 35 weeks of age, respectively. In contrast, the corresponding percentages of Program 2 gilts had a lower level of neutralizing antibody. Neutralizing antibody levels in Program 2 gilts were significantly higher after stimulation (p = 0.0004) and were detected in 15%, 80%, 70%, and 70% of gilts at 13, 17, 28, and 35 weeks of age, respectively.

## Discussion

In this study, we evaluated a PED outbreak using RT-qPCR and nucleotide sequencing methods to obtain genetic information about the virus. The results of the genetic analysis of PEDV indicate that all viruses identified in this study are 100% homologous. It should be noted that the PED outbreak in this farm might occur due to viral persistence in the farm. Then, the gilt acclimatization program was implemented in response to this outbreak and included monitoring PEDV shedding using RT-qPCR. Porcine epidemic diarrhea virus was detected before gut feedback in the gilts of both Programs, which can be explained by the possible exposure of the pigs to the virus circulating in wean-to-finish barns. Moreover, PEDV was detected continuously in Program 1 gilts, with some replacement gilts continuously transmitting PEDV during the replacement period. This PEDV strain was consistent with the same PEDV genetic results, and thus, it might have been the possible source of the circulation of PEDV in the breeding herd [12].

In Program 2, disease transmission was controlled in the replacement gilts before they entered into the herd. The modified Program 2 method involved early gut feedback and gilt flow management adjusted to reduce the chances of PEDV transmission within the farm. Continuous farming is a system that has been adjusted by the application of all-in/all-out replacement gilts, stringent disinfection procedures, and the adoption of biosafety measures. All of these measures, applied in Program 2, have controlled PEDV infection [10]. Interestingly, gut feedback implementation at the disease initiation stage has successfully minimized the outbreak period. However, it may not be able to terminate the shedding of PEDV within the population, thereby leading to the recurrence of PEDV infection within a year [18]. The duration of PED shedding typically ranges from 7 to 8 weeks [19]. Moreover, PEDV contamination persists on materials for up to 5 weeks at room temperature [20]. Therefore, gilts should not be exposed to gut feedback for a minimum of 10 weeks until replacement [11].

It is concerning that the immunity of replacement gilts can affect the lactogenic immunity that protects suckling piglets against PEDV and is conferred by maternal antibodies. Therefore, we compared the immune responses between the acclimatization methods in Programs 1 and 2. Some gilt in both programs developed a specific immune response to PED before immunization, which may be due to natural exposure to circulating PEDV before initiating gut feedback [21]. The immune response in Program 1 gilts increased after gut feedback and vaccination, while that in Program 2 gilts also increased after gut feedback. However, it tended to decline faster than that in Program 1. This may have been due to the incorrect timing of the booster vaccination in Program 2. Administration of gut feedback should adjust multiple feedbacks 2–3 times over 2 weeks. The goal of this method is to ensure that all sows are exposed to the virus [22].

Our results suggest that Program 2 is a suitable method to control PEDV circulation within the farm. However, the immunity level of the replacement gilts in Program 2 was lower than that of the replacement gilts in Program 1, and this may have been due to the implementation of an improper immunization method that failed to sufficiently immunize the pigs [23]. Our results also show that the administration of booster vaccination for PED during the prenatal period should consider lactogenic immunity levels. Therefore, future studies should evaluate the immunization method.

## Conclusion

Our study demonstrated that it was difficult to eliminate PEDV on the farm by practicing gut feedback, which tended to become a source of virus circulation and reinfection. Thus, a suitable acclimatization protocol should be applied for gilt immunization. Moreover, gilts should be provided with an appropriate cool-down period, from gut feedback to replacement, lasting at least 10 weeks. Nevertheless, our results suggest that the immune responses following immunization with gut feedback and vaccines may not provide adequate immune protection at replacement. Therefore, a comprehensive and appropriate acclimatization protocol and successful biosecurity management are needed to prevent and control PED effectively.

## Authors’ Contributions

PS, AB, and YW: Conceptualized and designed the study and prepared the initial draft of the manuscript. PS, SP, AB, and YW: Performed the sample collection, experiments, and data analysis. PS, MS, WC, OB, and KW: Conducted the RT-qPCR, ELISA, neutralization test and sequencing analysis. PS and PJ: Statistical analysis. YW: Finalized the manuscript. All authors have read, reviewed, and approved the final manuscript.
